# Accession-Dependent *CBF* Gene Deletion by CRISPR/Cas System in Arabidopsis

**DOI:** 10.3389/fpls.2017.01910

**Published:** 2017-11-07

**Authors:** Sungkyung Cho, Si-in Yu, Junghoon Park, Yanfei Mao, Jian-Kang Zhu, Dae-Jin Yun, Byeong-ha Lee

**Affiliations:** ^1^Department of Life Science, Sogang University, Seoul, South Korea; ^2^Department of Biomedical Science and Engineering, Konkuk University, Seoul, South Korea; ^3^Shanghai Center for Plant Stress Biology and Center of Excellence in Molecular Plant Sciences, Chinese Academy of Sciences, Shanghai, China; ^4^Department of Horticulture and Landscape Architecture, Purdue University, West Lafayette, IN, United States

**Keywords:** CRISPR, Cas, CBF, cold stress, cold signaling, accessions

## Abstract

The CRISPR/Cas system became a powerful genome editing tool for basic plant research and crop improvement. Thus far, CRISPR/Cas has been applied to many plants, including Arabidopsis, rice and other crop plants. It has been reported that CRISPR/Cas efficiency is generally high in many plants. In this study, we compared the genome editing efficiency of CRISPR/Cas in three different Arabidopsis accessions [Col-0, Ler, and C24RDLUC (C24 accession harboring the stress-responsive RD29A promoter-driven luciferase reporter)]. For the comparison, we chose to target the cold-responsive *C-repeat/DRE-Binding Factor* (*CBF*) genes. *CBF1*, *CBF2*, and *CBF3* genes are tandemly located on Arabidopsis chromosome 4 with redundant functions as the key transcription factors functioning in cold stress signaling and tolerance. Due to the close proximity of these *CBF*s on the chromosome, it is impossible to generate *cbf1, cbf2, cbf3* triple mutants (*cbf123*) by traditional genetic crosses. Therefore, using the CRISPR/Cas tool, we aimed to generate *cbf123* mutants and compared the genome editing efficiency in different Arabidopsis accessions. Among the accessions, Ler was the most resilient to the CRISPR/Cas deletion with the lowest gene deletion ratio in both T1 and T2 generations. Interestingly, while C24RDLUC showed a high *CBF123* deletion frequency in T2 only when the gene deletion was observed in T1 generation, Col-0 displayed high ratios of the *CBF123* deletions in T2 regardless of the presence or absence of the *CBF123* deletion in T1. Isolated *cbf123* mutants in C24RDLUC background showed no expression of *CBF1*, *CBF2*, and *CBF3* genes and proteins with reduction in the *CBF* target gene expression under cold stress.

## Introduction

The clustered regularly interspaced short palindromic repeats/CRISPR-associated (CRISPR/Cas) system is a new technology for targeted genome editing. The CRISPR/Cas system was first studied as an adaptive immune system for prokaryotes to defend themselves from foreign nucleic acids invasion (Wiedenheft et al., 2012; Sampson and Weiss, 2014). Although other targeted genome editing methods, such as transcription activator-like effector nuclease (TALENs) and zinc finger nucleases (ZFN), can generate genome modifications, the CRISPR/Cas system is a more affordable, robust, and easy genome editing tool (Mali et al., 2013; Shan et al., 2013; Sampson and Weiss, 2014). As CRISPR/Cas uses a guide RNA to specify the editing target DNA sequence, CRIPSR/Cas system does not need the elaborate design and assembly of DNA-binding proteins and makes it possible to generate a construct ready for transformation with a synthesis of simple DNA oligomers. The synthesized DNA oligomers are transcribed into single guide RNA (sgRNA) that guides the Cas9 DNA endonuclease to the target sites by sgRNA hybridization. The endonuclease Cas9 makes a double strand break at 3 bp upstream of Palindromic Adjacent Motif (PAM) sequence. The DNA breakage is repaired by homologous recombination (HR) or the error-prone non-homologous end joining (NHEJ) mechanism (Schiml et al., 2014). The NHEJ mechanism is known to be the major double strand break repair pathway in plants (Britt, 1999). During the NHEJ DNA repair process, the errors can be introduced causing irreversible mutations at the target sites in plants. In addition, multiple DNA breakages are possible in plants by introducing multiple sgRNAs to the target plants. These multiple DNA breakages can cause multiple mutations or large deletions depending on distance among the target sites (Li et al., 2013; Mao et al., 2013).

The CRISPR/Cas system works in different rates depending on the target region and the sequence of the sgRNA (Mali et al., 2013; Mao et al., 2013). Thus, in the present study, we aimed to test the efficiency of CRISPR/Cas in various Arabidopsis accessions - Col-0, Ler, and C24RDLUC (C24 accession with the stress-responsive RD29A promoter-driven luciferase reporter). We chose these accessions because Col-0 and Ler are among the most commonly used ones in Arabidopsis and C24RDLUC would make it easy to examine CBF-target gene down-regulation by using luciferase imaging system (Ishitani et al., 1997). As genome editing target genes, we chose to delete C-repeat/DRE-Binding Factor (*CBF*) genes that are important in cold stress signaling in plants. Upon cold stress, plants increase the expression of *Cold Regulated* (*COR*) genes that molecularly adapt the plant to withstand cold stress (Chinnusamy et al., 2007; Park et al., 2015). The key signaling pathway for the expression of *COR* genes is the *CBF* signaling pathway. *CBF*s are transcription factors with a conserved DNA-binding domain found in the ethylene-responsive element-binding factors (ERF) and floral homeotic protein APETALA 2 (AP2) proteins. In Arabidopsis genome, there are four *CBF* genes. Among them, *CBF1, CBF2*, and *CBF3* are early induced by cold, but not by drought and salt stresses (Yamaguchi-Shinozaki and Shinozaki, 2006). By contrast, *CBF4* gene expression is up-regulated by drought stress, but not by low temperature (Haake et al., 2002). Accordingly, *CBF1*, *CBF2*, and *CBF3* (*CBF123* hereafter when all the three genes are mentioned) function as primary transcription factors for cold tolerance. *CBF1*, *CBF2*, and *CBF3* are closely aligned within 7.1K base pairs on the chromosome 4 of Arabidopsis with a small intergenic distance (2–3 Kbp). Due to this tandem array of the three genes, it is almost impossible to generate *cbf123* triple mutants by traditional crossings. However, with the emergence of the CRISPR/Cas system, generation of *cbf123* triple mutants has become possible by targeting the tandemly located all three *CBF* genes. Indeed, very recently, four lines of *cbf123* triple mutants were reported in Columbia-0 background (Jia et al., 2016; Zhao et al., 2016; Zhao and Zhu, 2016).

In this study, we found that Ler showed the lowest ratio of the *CBF123* gene deletion by CRISPR/Cas in both T1 and T2 generations. In addition, Col-0 and C24RDLUC displayed generally high ratios of the *CBF123* gene deletion in T1 and T2 generation. Interestingly, the high ratios of the *CBF123* deletions in Col-0 were observed regardless of the presence or absence of the gene deletion tested in the leaves of each T2’s progenitor (T1) while C24RDLUC showed a high *CBF123* deletion frequency in T2 generation when high gene deletion ratios were observed in T1 generation. Isolated *cbf123* mutants in C24RDLUC background (*cbf123LUC-2*) showed no expression of *CBF1*, *CBF2*, and *CBF3* genes and proteins were detected in these *cbf123LUC-2* mutants after cold treatment, suggesting that *cbf123LUC-2* is a null mutant. Accordingly, *CBF* target gene expression in *cbf123LUC-2* was reduced under cold stress in comparison with its background wild type. *cbf123LUC-2* displayed a smaller size than wild type at the early development stage.

## Materials and Methods

### Plant Growth

Arabidopsis seeds were surface-sterilized with bleach (∼4% sodium hypochlorite) and plated on MS plates. After plating, the plates were kept at 4°C for at least 2 days to obtain germination synchrony before being transferred to 22°C under constant illumination (80–100 μmol m^-2^S^-1^) and 70% relative humidity for germination and growth. Murashige and Skoog (MS) medium (pH 5.8) was made with full strength MS salts (Caisson Laboratories, United States), 2% sucrose, and 0.3% gelite (Duchefa, Netherlands). For selection plates, hygromycin B was added to a final 25 mg/L concentration to the MS media. For soil growth, the seeds planted on soil (Sungro mixture#5, Canada) were placed in a growth room operating at 22°C with the cycle of 16-h of light and 8-h of darkness (the light intensity of 80–100 μmol m^-2^S^-1^) after 2 days of cold stratification.

### CRISPR/Cas9 Construct Generation

For the selection of multiple targeting sgRNA, multiple sequence alignment software Clustal W^1^ was used to align the coding sequence of *CBF1*, *CBF2*, and *CBF3*. Two 19-bp sequences (sgRNA12 and sgRNA23) immediately before a PAM sequence (5′-NGG-3′) were selected and used for DNA oligomer synthesis for sgRNA.

The forward and reverse DNA oligomers for sgRNA targeting *CBF1* and *CBF2* were CBF12-sgR1-F (5′-GATTGAGCTGCCATCTCAGCGGTT-3′), CBF12-sgR1-R (5′-AAACAACCGCTGAGATGGCAGCTC-3′) and for sgRNA targeting *CBF2* and *CBF3* were CBF23-sgR1-F (5′-GATTGGAGTCAGCGAAATTGAGAC-3′) and CBF23-sgR1-R (5′-AAACGTCTCAATTTCGCTGACTCC-3′).

Following the protocol suggested by Liu et al. (2015), psgR-Cas9-At was used to generate each single sgRNA-containing vector (i.e., sgRNA12-Cas9 vector and sgRNA23-Cas9 vector). To make a double sgRNA-containing sgRNA12-sgRNA23-Cas9 vector, sgRNA23 module from the sgRNA23-Cas9 plasmid was PCR-amplified using a following primer pair (sgR_U6_Kpn1-F, 5′-GCCGGTACCCATTCGGAGTTTTTGTAT-3′; sgR_end_EcoRI-R, 5′-TATGAATTCGCCATTTGTCTGCAGAATTG-3′). The resultant PCR product was inserted into the KpnI and EcoRI sites of sgRNA12–Cas9 vector. Finally, the whole cassette of sgRNA12-Cas9-sgRNA23 released by HindIII and EcoRI from the double sgRNA containing construct was subcloned to the HindIII-EcoRI sites of pCAMBIA1300, resulting in the pCAMBIA-sgRNA12-sgRNA23-Cas9 construct. A schematic drawing of the construct generation is shown in **Supplementary Figure S1**.

### Construction of CRISPR/Cas Transgenic Arabidopsis and Detection of *cbf123* Deletion

*Arabidopsis thaliana* plants (accession Col-0, Ler, and C24RDLUC) were transformed with pCAMBIA-sgRNA12-sgRNA23-Cas9 construct via *Agrobacterium tumefaciens* strain GV3101 by floral dipping (Clough and Bent, 1998). T1 seeds were collected from the floral dipped plants and then selected on MS plates with hygromycin B 25 μg/mL. The selected T1 plants were transferred to soil and genotyped with 3 different primer pairs (pCAM1300intC-R, 5′-GGCCTCTTCGCTATTACGC-3′ and CBF12-sgR1-R, 5′-AAACAACCGCTGAGATGGCAGCTC-3′ for sgRNA12; doublesgRNA-F, 5′-GATCGACCTGTCTCAGCTGG-3′ and pCAM1300intC-F, 5′-ATTAATGCAGCTGGCACGAC-3′ for sgRNA23; Cas9-F, 5′-CCCAACTTCAAGAGCAACTT-3′ and Cas9-R, 5′-TCACTTTGGTCAGCTCGTTA-3′ for Cas9). For the *cbf123* deletion detection, nested PCR was employed with the following primer pairs were used (CBF1-PS-F, 5′-CGTGTGCTCCCCACATATC-3′ and CBF2-PS2-F, 5′-ATTTGTTGCTTATGGGGAGA-3′ for the first round PCR; CBF1_small_R, 5′-AATCCAAAAAGACTGAGAACTCTA-3′ and CBF2q-R, 5′-CTGCACTCAAAAACATTTGCA-3′ for the second round PCR). For the *cbf123* deletion confirmation and homozygosity test, 4 different primers were used (CBF1_samll_R, CBF2q-R, CBF1-PS-R, 5′-CCGCTTTTTGGATATCCTTG-3′ and CBF2qRT-F(172), 5′-AACTCCGGTAAGTGGGTGTG-3′).

### Luminescence Imaging

Luminescence intensities of *cbf123LUC-2* were measured by the Lumazone luminescence imaging system (Roper Scientific, United States). Ten-day-old seedlings grown on MS agar plates were incubated at 0°C for the designated times and the luminescence images were taken after luciferin spray. The luminescence intensity of each seedlings was quantified with the WinView32 program.

### Gene Expression Analysis

Total RNA was extracted from 11- to 13-day-old seedlings with or without cold (0°C) treatment using the RNAiso Plus reagent (Takara, Japan). After DNase I treatment (New England Biolabs, United States), cDNA was synthesized with 5 μg total RNA using the TOPScript^TM^ Reverse Transcriptase Kit (Enzynomics, Korea). cDNA was used as a template for quantitative real-time PCR (qRT-PCR) using SYBR^®^ FAST (KAPA Biosystems, United States) on the LightCycler^®^96 (Roche, Switzerland). The following primer pairs were used for real-time PCR (CBF1q-F, 5′-GCATGTCTCAACTTCGCTGA-3′ and CBF1q-R, 5′-ATCGTCTCCTCCATGTCCAG-3′ for *CBF1*; CBF2q-F, 5′-TGACGTGTCCTTATGGAGCTA-3′ and CBF2q-R, 5′-CTGCACTCAAAAACATTTGCA-3′ for *CBF2*; CBF3q-F, 5′-GATGACGACGTATCGTTATGGA-3′ and CBF3q-R, 5′-TACACTCGTTTCTCAGTTTTACAAAC-3′ for *CBF3*; RD29A_RT-F, 5′-CTTGTCGACGAGAAGCAAAGAA-3′ and RD29A_RT-R, 5′-TCTTGATGGAGAATTCGTGTCC-3′ for *RD29A*; COR15A_RT-F, 5′-ACTCAGTTCGTCGTCGTTTCTC-3′ and COR15A_RT-R, 5′-TCTCACCATCTGCTAATGCCTC-3′ for *COR15A*; COR47_qRT-F, 5′-TGTCATCGAAAAGCTTCACCGA-3′ and COR47_qRT-R, 5′-ACCGGGATGGTAGTGGAAACTG-3′ for *COR47*; KIN1_qRT-F, 5′-ATGCCTTCCAAGCCGGTCAGAC-3′ and KIN1_qRT-R, 5′-CCGGTCTTGTCCTTCACGAAGT-3′ for *KIN1*; Clathrin-F, 5′-CTGACTGGCCCTGCTT-3′ and Clathrin-R, 5′-ATACGCGCTGAGTTCCC-3′ for Clathrin as the internal control).

### Protein Blot Analysis

For protein blot analysis for CBF123 protein detection, 14-day-old seedlings of wild type and *cbf123LUC-2* on MS agar plates were cold-treated at 0°C for 6 h, the seedlings were frozen-ground and suspended in protein sample buffer [130 mM Tris-Cl (pH 8.0), 4.6% (w/v) SDS, 0.02% Bromophenol blue, 2% DTT, 20% (w/v) Glycerol]. Samples were boiled for 3 min and centrifuged at 13,200 rpm for 10 min at 4°C. The supernatant was run on the sodium dodecyl sulfate polyacrylamide gel electrophoresis (SDS-PAGE) gel (Nguyen et al., 2016). The resultant gel was blotted on to the polyvinylidene fluoride (PVDF) membrane and the existence of CBF proteins were detected with rabbit anti-CBF123 antibody and anti-rabbit goat anti-rabbit IgG-HRP conjugate (1:10000, Abcam, United Kingdom). Anti-CBF123 antibody was raised with full length protein of CBF2. Further detail on the method of anti-CBF123 generation is provided in **Supplementary Figure S3**.

### Statistical Analysis

Statistical analyses of the results from the experiments of three accessions were performed using two-way analysis of variance with Microsoft Excel program (2010 version), and significant differences between each index among accessions and among T2 ratios of the same accession were determined using Student’s *T* test.

## Results

### Small Guide RNA Sequence Selection for *CBF1*, *CBF2* and *CBF3*

**FIGURE 1:**
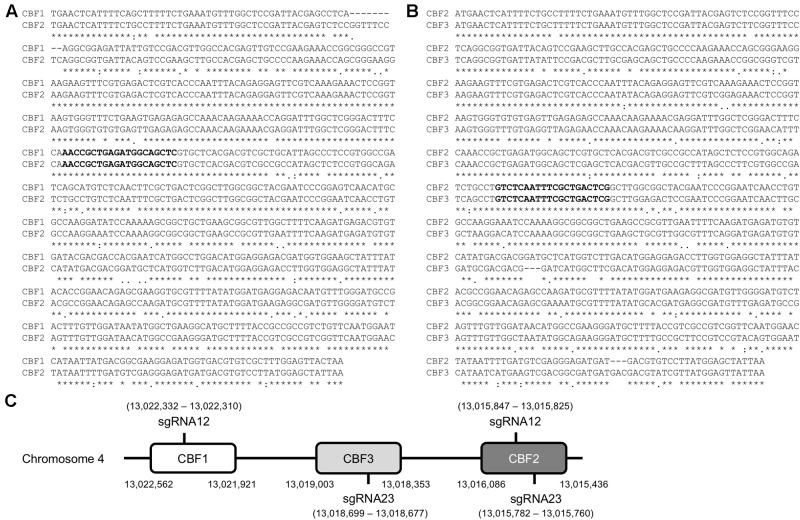
Design of DNA oligomer for sgRNA targeting *CBF1*, *CBF2* and *CBF3.* **(A**,**B)** Alignments of coding sequences of *CBF1/CBF2* **(A)** and *CBF2/CBF3* **(B)**. The bold sequences denote the selected sgRNA sequence and ^∗^indicates the same sequence between *CBF*s. **(C)** Schematic drawing of the location of DNA oligomer for sgRNAs targeting *CBF1*, *CBF3*, and *CBF2* as in the order on the chromosome 4 of *Arabidopsis thaliana*. Numbers in parenthesis indicate the nucleotide numbers of chromosome 4.

To generate a triple mutant of *CBF1*, *CBF2*, and *CBF3* genes using the CRISPR/Cas system, two possible sgRNA target sites were selected by aligning *CBF1* and *CBF2*, and *CBF2* and *CBF3* with a multiple sequence alignment program (Clustal W)^2^ (**Figures 1A,B**). The two sgRNA target sequences were named sgRNA12, which targets *CBF1* and *CBF2*, and sgRNA23, which targets *CBF2* and *CBF3* (**Figures 1A–C**). There were no single sgRNA site that could target all three CBF genes in the coding sequence (CDS) region at the same time. By using these two sgRNAs, the CRISPR/Cas system can target the three *CBF*s in the four sites (**Figure 1C**). Since the sgRNAs will be used in three different accessions, Col-0, C24RDLUC and Ler, the *CBF* regions of these accessions were sequenced. We found that the DNA sequences for sgRNA12 and sgRNA23 were identical in all three accessions (data not shown). Then, the possible off-target sites of sgRNA12 and sgRNA23 were analyzed by the online program CRISPR-P^3^ (Lei et al., 2014). The analysis revealed that the off-target possibility of both sgRNA12 and sgRNA23 was extremely low (scores of 0.0–5.7 for off-target sites vs. 100 for *CBF1*, *CBF2*, and *CBF3* genes), suggesting that each would very specifically target *CBF1*/*CBF2* and *CBF2*/*CBF3*, respectively (**Table 1**).

**Table 1:** Selected DNA sequence for sgRNA and possible off-target sequence analyzed by CRISPR-P program.

	Sequence	Score	AGI ID	Gene name
sgRNA12	GAGCTGCCATCTCAGCGGTTTGG	100	AT4G25480	CBF2
	GAGCTGCCATCTCAGCGGTTTGG	100	AT4G25470	CBF1
	GAGCAGCCATGTCAGGGGCTTGG	0	AT3G17410	Unknown protein
	GCGCTGCCATCTCCGCCGTGGGG	0	AT2G25820	ESE2
sgRNA23	CGAGTCAGCGAAATTGAGACAGG	100	AT4G25480	CBF2
	CGAGTCAGCGAAATTGAGACAGG	100	AT4G25470	CBF3
	AGAATCAGCGAAATTGAGACAAG	5.7	AT5G51990	CBF4
	CGTTTCAGCGAAATTGATAAGGG	0.1	AT2G47790	GTS1
	CGAGACTGAGAAATTTAGACGGG	0.1		Intergenic region


### Identification of T1 Seedlings with a Complete Set of Transgenes

In order to target *CBF1*, *CBF2*, and *CBF3* genes, DNA oligomers for sgRNA12 and sgRNA23 were inserted into psgR-Cas9-At vector containing the Cas9 sequence with a chimeric RNA backbone for the sgRNA (Feng et al., 2013; Mao et al., 2013; Liu et al., 2015). The whole cassette with Cas9 was then subsequently subcloned into pCAMBIA1300 to generate the CBF targeting CRISPR/Cas binary vector construct. The construct was used to transform three Arabidopsis accessions through *Agrobacterium* mediated plant transformation. The seeds from the floral-dipped plants (T1 seeds) were harvested and plated on hygromycin plates for the positive transformant selection. The presence of transgenes including sgRNA12, sgRNA23, and Cas9 in the positive T1 seedlings was then tested by PCR with the primers used for construct component confirmation. While generally high (75–100%), the detection ratios of each transgene in each accession varied, implying various occurrences of the whole transgene insertion in each accession (**Table 2**). The ratios of plants with a complete set of three transgenes were higher than 60% in all accessions with Columbia-0 being the highest (80%) (**Table 2**). These transgenic plants with a whole transgene set were further analyzed for CRISPR/Cas efficiency on the *CBF* genes.

**Table 2:** Detection ratio of the sgRNA12-sgRNA23-Cas9 transgenes in T1 plants.

Accession	sgRNA12	Cas9	sgRNA23	sgRNA12 and Cas9	Cas9 and sgRNA23	sgRNA12 and sgRNA23	sgRNA12, Cas9 and sgRNA23
Col-0	95.00	100.00	95.00	95.00	95.00	90.00	16/20 ^∗^ 80.00%
C24RDLUC	84.00	84.00	94.00	72.00	80.00	82.00	33/50 ^∗^ 66.00%
Ler	87.50	89.58	87.50	81.25	81.25	75.00	35/48 ^∗^ 72.92%


*Detection ratio (%). ^∗^Number of plant with all transgenes of sgRNA12, Cas9 and sgRNA23/total number of plants examined.*

### Accession-Dependent *CBF123* Deletion Efficiency in T1 Plants

**FIGURE 2:**
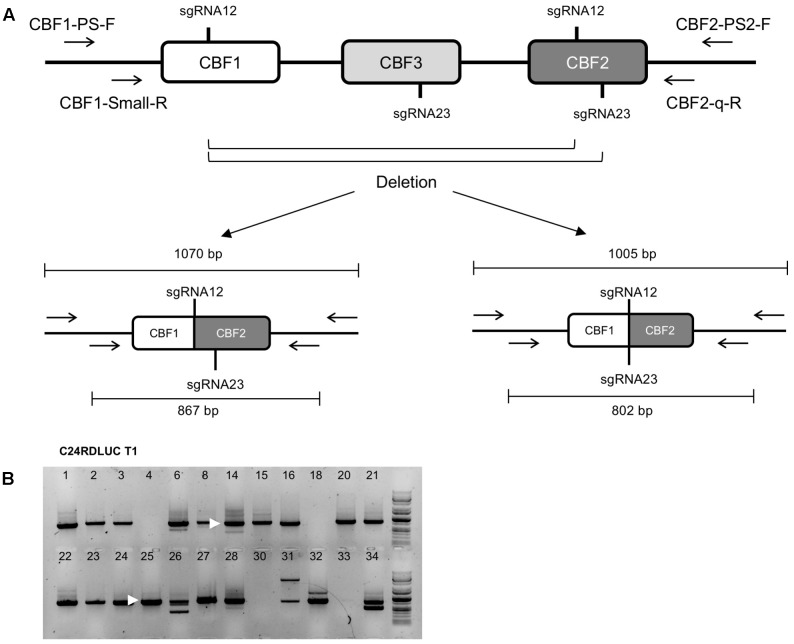
Detection of large deletion for *cbf123* triple mutation in T1 plants. **(A)** Schematic drawing of *CBF123* deletion detection. Possible cut sites of sgRNA between sgRNA12 sites of *CBF1* and *CBF2* will generate a 1070 bp band and those between sgRNA12 of *CBF1* and sgRNA23 of *CBF2* will generate a 1005 base pair band. **(B)** Nested PCR results to detect a large deletion for *cbf123* triple mutation. Arrow heads indicate the band sizes about 800 bp.

We then asked in T1 generation if our sgRNAs were functional in CRISPR/Cas-mediated genome editing and in what ratio the CRISPR/Cas-caused *CBF123* deletions would occur in each accession. Our sgRNA design intended to target four sites (two sites per sgRNA) in *CBF1*, *CBF2*, and *CBF3* genes (**Figure 1C**) to maximize the gene editing efficiency. Among the deletions that would be produced by our CRISPR/Cas system, the deletions for the *cbf123* triple mutation should occur between the sgRNA12 site on *CBF1* and sgRNA12 or sgRNA23 site on *CBF2* (**Figure 1C**). In order to detect these deletions of the *CBF* gene region, we performed PCR using a primer set of one primer aligning to the 5′ UTR region of *CBF1* and the other aligning at the 3’ UTR region of *CBF2*. Ca. 1070 bp and/or 1005 bp PCR products were expected if the deletion for *cbf123* mutation took place (**Figure 2A**). Our initial PCR produced only several faint bands. It is highly likely because of the low numbers of cells with *cbf123* deletion mutation in these T1 plants. It should be noted that T1 plants would contain heterogeneous cells with various CRISPR/Cas effects. Therefore, nested PCR was conducted with the 1000 times diluted initial PCR products. Nested PCR would also ensure that the faint bands are not non-specific and that the bands are also sufficiently intensified to be visible. As expected, in the nested PCR results, even the samples without initial visible bands displayed clear bands (**Figure 2B**). Among the T1 plants with all transgenes, the large deletion ratios for *cbf123* triple mutations varied between 45.71 and 84.85% among the accessions with Ler being the lowest and C24RDLUC the highest (**Table 3**). These results suggest that the CRISPR/Cas system has accession-dependent efficiency in the generation of *CBF123* deletion.

**Table 3:** *CBF123* deletion ratio among T1 plants harboring all transgenes of sgRNA12, Cas9 and sgRNA23.

Accession	sgRNA12, Cas9 and sgRNA23^∗^	Large deletion mutation^∗∗^
Col-0	16/20 (80.00%)	12/16 (75.00%)
C24RDLUC	33/50 (66.00%)	28/33 (84.85%)
Ler	35/48 (72.92%)	16/35 (45.71%)


*^∗^Number of plants with all transgenes of sgRNA12, Cas9 and sgRNA23/total number of plants examined. ^∗∗^Number of plants with *CBF123* deletion/number of plants with all transgenes of sgRNA12, Cas9 and sgRNA23.*

### Various *CBF123* Deletion in T1 Plants

**FIGURE 3:**
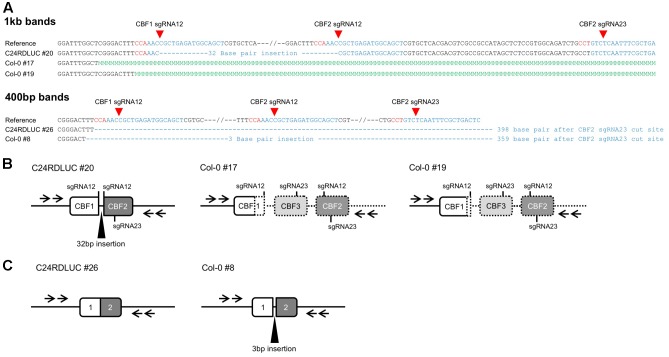
Various mutations found in T1 plants. **(A)** Alignments of sequences of the PCR product from T1 plants and the reference sequence. Red sequences indicate the PAM sequence, blue sequences denote the sgRNA sequences and the green “M” sequences mean multiple peaks shown in sequence electropherogram. **(B)** Drawing of the *CBF123* deletion that will give rise to about 1 kb product from PCR with primers indicated by arrows. The drawings in dotted line indicate DNA regions with multiple sequencing peaks due to genetically mosaic T1 leaf tissues. **(C)** Drawing of the *CBF123* deletion that will give rise to about 400 bp product from PCR with primers indicated by arrows.

To determine whether the deletion occurred in the sgRNA cut sites, the nested PCR products from T1 plants were sequenced. Although the sizes of the PCR bands varied considerably, most bands showed a size of ca. 800 base pairs, which was in agreement with the assumption that the *CBF123* deletion mutations would occur at the sgRNA cut sites present on *CBF1* and *CBF2* (**Figure 2**). CRISPR/Cas action can result in mutations with a few nucleotide additions or deletions at the sgRNA target site due to the error-prone non-homologous end joining pathway (Belhaj et al., 2013; Hsu et al., 2014; Lozano-Juste and Cutler, 2014; Liu et al., 2015). Because of this, T1 plants become genetically mosaic with cells containing different mutations at the sgRNA target sites. Thus, the PCR products similar in size could be heterogeneous and the sequencing of the PCR products could produce multiple peaks at the nucleotide after the sgRNA cut sites. Our sequencing results showed that the deletion started at the cut site of sgRNA or in close proximity to it, implying a specific double strand break on the target sites (**Figures 3A,B**). Our results also reveal that addition or deletion of nucleotides at the sgRNA target also occurred in our *CBF123* deletion mutations (**Figures 3A,B**). Occasionally, we observed the sequencing results from some unexpected size products (**Figures 3A,C**). These could be due to the prolonged DNA repair which might lead to extra base pair deletion after the sgRNA cut site (Gorbunova and Levy, 1999).

### Accession-Dependent *CBF123* Deletion Inheritance to T2 Plants

**FIGURE 4:**
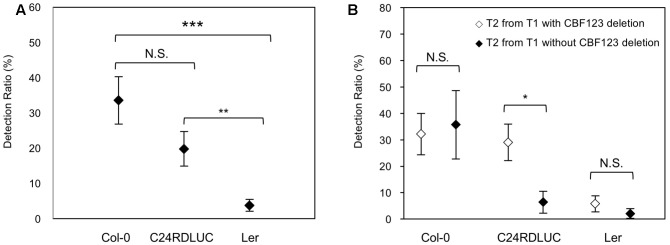
Accession-dependent *CBF123* deletion inheritance to T2. **(A)** Averages of each accession’s *CBF123* deletion ratio in T2 plants. **(B)** Averages of each accession’s *CBF123* deletion ratio in T2 plants from T1 with *CBF123* deletion or without *CBF123* deletion. Asterisks indicate a significant difference (^∗^*p* < 0.05, ^∗∗^*p* < 0.01, ^∗∗∗^*p* < 0.001, N.S., non-significant). The error bars indicate the standard deviation.

The T2 seeds from the T1 plants were harvested and used for the analysis of genetic inheritance of the *CBF123* deletion and the *cbf123* triple mutant screening. We examined T2 progenies from all T1 plants with a complete set of sgRNAs and Cas9 transgenes; T2 progenies from 16 T1 Col-0 plants, 33 T1 C24RDLUC plants, and 35 T1 Ler plants were analyzed (**Table 3** and Supplementary Tables S1–S3). In most cases, we analyzed 24 T2 seedlings per T1 line. We found that *CBF123* gene deletions could be observed in T2 seedlings not only from the T1 lines with the *CBF123* deletion, but also from the T1 lines without the deletions. Also, there were some cases when the *CBF123* gene deletion was not inherited to T2 seedlings from the T1 plants that contained the *CBF123* deletions (Supplementary Tables S1–S3). This indicates that the CRISPR/Cas9-induced mutations in germ cells do not always occur even when the *CBF123* deletion in somatic cells (leaf cells) exists at T1. Interestingly, the transmission ratios of *CBF123* deletion from T1 to T2 generation were different among the Arabidopsis accessions. Although not significantly different, the ratios of the *CBF123* deletion at the T2 generations in Col-0 was the highest (33.56%) followed by C24RDLUC (19.80%). Ler showed the lowest *CBF123* gene deletion ratio (3.74%) (**Figure 4A**). We also noticed the different accession-dependent inheritance ratios of the *CBF123* deletion among the T2 progenies from T1 lines with and without the *CBF123* deletions (**Figure 4B**). In Col-0, the *CBF123* deletions were detected at very similar ratios in the T2 progenies, regardless of the presence of *CBF123* deletions at the T1 generation (32.20% in T2 from *CBF123*-deleted T1 vs. 35.71% in T2 from *CBF123* not-deleted T1) (**Figure 4B**). By contrast, C24RDLUC showed a higher transmission ratio of the *CBF123* deletions in T2 lines from T1 lines with the deletion rather than in those from T1 lines without the deletions (29.0% vs. 6.40%) (**Figure 4B**). Though similar in the *CBF123* deletion ratios in either T2 lines of Ler (5.77% vs. 2.08%), Ler showed very low *CBF123* deletion ratios in T2 (**Figure 4B**).

### Isolation of Homozygous *cbf123* Triple Mutants in the T2 Generation

**FIGURE 5:**
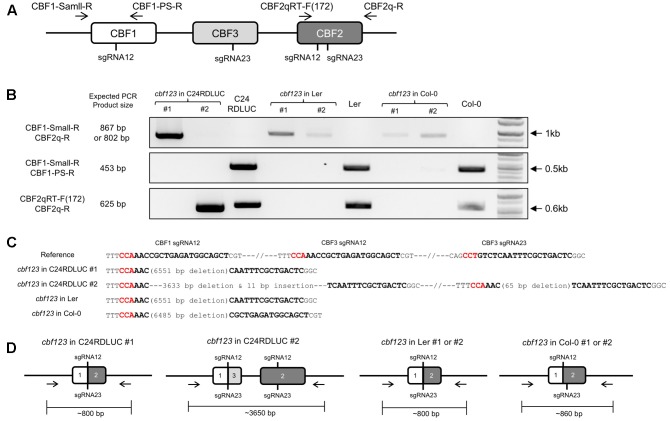
Homozygous *cbf123* triple mutant generated by CRISPR/Cas system. **(A)** Alignment of the four primers used for homozygous *cbf123* mutant detection. **(B)** *CBF123* deletion confirmation. **(C)** Alignment of sequences of the PCR product from the homozygote mutants in C24RDLUC, Ler and Col-0 backgrounds and the reference sequence. Red sequences indicate the PAM sequence, bold sequences indicate the sgRNA sequences. **(D)** Schematic diagram of the homozygous *cbf123* mutants in each accession.

In order to isolate the homozygote mutants, we analyzed homozygosity of the candidate *cbf123* triple mutants in each accession by PCR with three pairs of primers that were designed to detect the combination of *CBF123* deletions (**Figure 5A**). Through the intensive PCR screening, we identified several homozygote mutants for the *CBF123* deletion in each accession (**Figure 5B**).

In the case of the C24RDLUC background *cbf123* mutant, we found two different homozygous mutants. *cbf123#1* in C24RDLUC (hereafter *cbf123LUC-1*) had a 6,551 bp deletion between the cut sites of sgRNA 12 and sgRNA 23 in *CBF2* while *cbf123#2* in C24RDLUC (hereafter *cbf123LUC-2*) contained two deletions; one deletion was a 3,633 bp deletion between the cut sites of *CBF1* sgRNA 12 and *CBF3* sgRNA23 with an 11 bp insertion, and the other was a 65 bp deletion between the cut sites of sgRNA12 and sgRNA23 of the *CBF2* region (**Figures 5C,D** and **Supplementary Figure S2**). Ler background *cbf123* mutant had a 6,551 bp large deletion between the cut sites of *CBF1* sgRNA 12 and *CBF2* sgRNA 23 and Col-0 background *cbf123* mutant showed a 6485 bp large deletion between the cut sites of *CBF1* sgRNA 12 and *CBF2* sgRNA 12 (**Figures 5C,D** and **Supplementary Figure S2**). In addition, no mutation was found in the potential off-target region of a closely related *CBF* paralog, the *CBF4* gene (data not shown).

**FIGURE 6:**
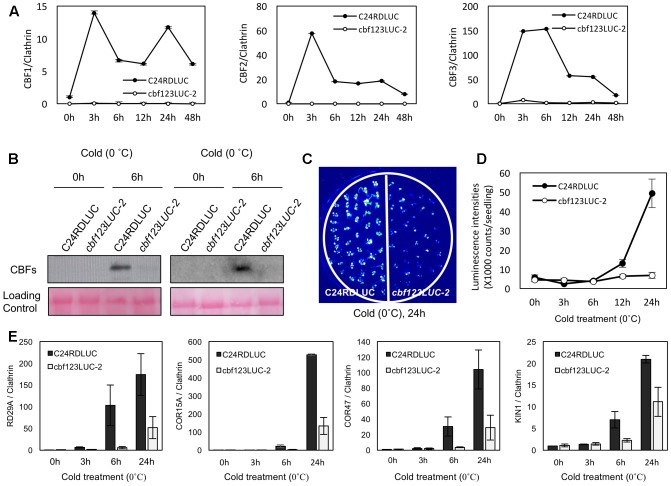
Gene expression in *cbf123LUC-2* mutant. **(A)** Expression of endogenous *CBF1*, *CBF2* and *CBF3* after cold treatment (0°C). 10-day-old seedlings were cold-treated for designated time before qRT-PCR analysis **(B)** Protein blots analysis to detect CBF proteins in 14-day-old seedlings using anti-CBF123 antibody with or without cold treatment (0°C, 6 h). Two biological repeats were shown. **(C)** Luminescence imaging of C24RDLUC and *cbf123LUC-2* after cold treatment (0°C, 24 h). **(D)** Luminescence intensities of C24RDLUC and *cbf123LUC-2* during cold treatment. **(E)** Expression of *RD29A*, *COR15A*, *COR47*, and *KIN1* after cold treatment (0°C). 10-day-old seedlings were cold-treated for designated time before qRT-PCR analysis. For qRT-PCR quantification, clathrin was used as the internal control. Error bars indicate standard errors of the three biological replicates.

**FIGURE 7:**
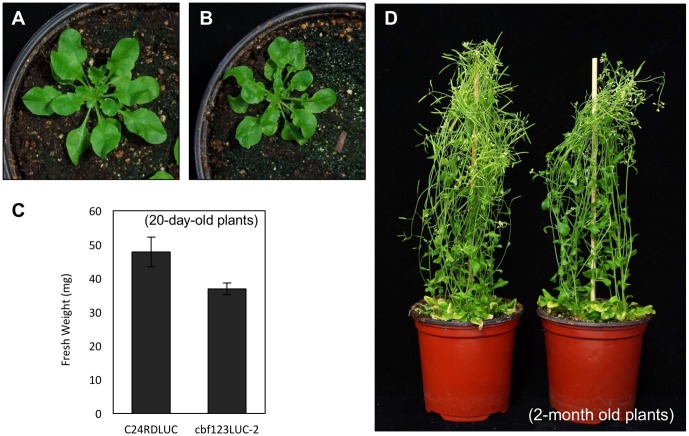
Growth of C24RDLUC and *cbf123LUC-2* plants. **(A,B)** Rosette leaf development of C24RDLUC **(A)** and *cbf123LUC-2* **(B)** plants (20-day-old plants). **(C)** Fresh weights of C24RDLUC and *cbf123LUC-2* (20-day-old plants). **(D)** Mature plants of C24RDLUC (left) and *cbf123LUC-2* (right) plants (2-month old plants).

Among these *cbf123* triple mutants, we decided to further characterize the *cbf123LUC-2* because this mutant’s background line, C24RDLUC, contains a stress-inducible *RD29A* promoter-driven luciferase, which will be beneficial in monitoring the *CBF* target gene expression *in vivo*. In addition, the Cas9 transgene was segregated out of *cbf123LUC-2*, but not in other *cbf123* triple mutants in other accession backgrounds. RNA transcripts and protein blot analysis confirmed the lack of the CBF proteins in *cbf123LUC-2*, indicating that *cbf123LUC-2* is a null mutant (**Figures 6A,B**). Luminescence imaging after cold treatment showed clear reductions of the RD29A-LUC luminescence in *cbf123LUC-2* in comparison to C24RDLUC (**Figures 6C,D**). This *RD29A-LUC* expression patterns were well correlated with those of the endogenous *RD29A* expression in *cbf123LUC-2* (**Figure 6E**). Additionally, expressions of other *CBF123* target genes were also reduced in *cbf123LUC-2* (**Figure 6E**). In growth and development, *cbf123LUC-2* appeared slightly smaller than C24RDLUC (**Figures 7A–C**) in early development stages. At the fully grown stage, there appeared to be no big differences in growth and development between C24RDLUC and *cbf123LUC-2* (**Figure 7D**).

## Discussion

In the present study, we found that CRISPR/Cas efficiency and inheritance could vary depending on Arabidopsis accessions. In the T1 generation, CRISPR/Cas efficiency was high in Col-0 and C24RDLUC with respective ratios of 75.00 and 84.85%, while it was only 45.71% in Ler (**Table 3**). *CBF123* deletions were also observed differently in the T2 generation depending on Arabidopsis accessions. In T2, both Col-0 and C24RDLUC showed non-significantly different ratios of the *CBF123* deletions, each displaying 33.56 and 19.80%, respectively (**Figure 4A**). However, the *CBF123* deletion frequency was only 3.74% in Ler (**Figure 4A**). These results suggest that CRISPR/Cas efficiency is very low in Ler at least for *CBF123* deletion. Interestingly, when T2 progenies from T1 with and without *CBF123* deletions were separately analyzed, only C24RDLUC showed a correlation between T1 and T2 generations in *CBF123* deletion frequencies (**Figure 4B**); T2 lines from T1 with *CBF123* deletions showed a higher deletion ratio than T2 lines from T1 without *CBF123* deletions. By contrast, Col-0 showed similar *CBF123* deletions in T2 regardless of the frequency of *CBF123* deletions at T1 (**Figure 4B**). Again, Ler T2 lines from T1 without *CBF123* deletions showed an extremely low deletion ratio (2.08%). Thus, practically, if one uses Col-0, the most commonly used Arabidopsis accession, one should consider our observation that both T1 lines with and without the CRISPR/Cas target gene mutation could produce the target gene mutation in the T2 generation. Accession-dependent CRISPR/Cas efficiency and inheritance of its targeted mutation suggest that susceptibility to CRISPR/Cas system could vary among the naturally variant Arabidopsis accessions. Ler was the most resilient to CRISPR/Cas-mediated *CBF123* deletion among the accessions tested. It showed the lowest *CBF123* deletion ratio of 45.71% at T1 and a 3.74% deletion ratio at T2 (**Figure 4A** and **Table 3**).

CRISPR/Cas-induced gene modification can only be transmitted through the germ cells. Indeed, it has been reported that germ cell or egg cell-specific expression of CRISPR/Cas resulted in less or no-mosaic T1 plants with enhanced ratios of transmittable mutation (Wang et al., 2015; Mao et al., 2016). Thus, it is feasible to assume that Ler is in general not susceptible to CRISPR/Cas editing and that the activities of CRISPR/Cas in germ cells of Ler are very low.

It was also interesting that Col-0 displayed a high *CBF123* deletion ratio at T2 regardless of the presence of *CBF123* deletions in the parental lines (T1) of the T2 progenies, while C24RDLUC showed a strong correlation of *CBF123* deletion ratio at T2 with *CBF123* deletion ratio at the parental T1 (**Figure 4B**). *CBF123* deletion at T1 are somatic mutations as genotyping was analyzed with the genomic DNA from young leaves of 15-day-old seedlings. Therefore, it might be possible that the CRISPR/Cas system is relatively highly active in the Col-0 germ cells independently of functional activation in somatic cells. By contrast, it might also be possible that there are not many differences in the CRISPR/Cas activity between somatic cells and germ line cells of C24RDLUC.

The differences among these accessions imply the presence of accession-specific genetic modifiers. The presence of such accession-specific modifiers is not uncommon. In particular, the mutation in *ZWILLE*/*PINHEAD*/*AGO10* (*ZLL*) shows defects in maintenance of embryonic shoot apical meristem. However, these defects were apparent in Ler, but not in Col-0, because the defects were masked by the modifiers in Col-0 (Tucker et al., 2013). One of the modifiers in the *ZLL* function appeared to be the Arabidopsis Cyclophilin-40 orthologue SQUINT (SQN, AT2G15790) (Tucker et al., 2013). In addition, the *altered meristem program* (*amp1*) mutation differently affected the flowering time in Col-0 and Ler (Lee, 2009), presumably because of the different genetic modifiers in these accessions. Similarly, accession-dependent CRISPR/Cas efficiency and inheritance could be due to the different modifiers in these accessions. The candidate genetic modifiers could include the genes involved in DNA repair as CRISPR/Cas-induced mutations rely on error-prone DNA repair. The natural variations in these gene structures and regulation might contribute to these differences in CRISPR/Cas action in Arabidopsis accessions.

Another possibility for these differences is that Cas9 access to locus of *CBF123* might be different in these accessions. It was shown that, in human cells, Cas9 access to the target sites was hindered by closed chromatin and restored upon induction of open chromatin status (Daer et al., 2017). In Arabidopsis, it has been known that the patterns of DNA methylation (hence the chromatin structure) is strongly correlated with the climate of accessions’ origin (Kawakatsu et al., 2016). Given that *CBF1, 2*, and *3* genes are cold-inducible, these Col-0, C24RDLUC, and Ler might have dynamic and different chromatin status affecting the efficiency of the CRISPR/Cas system. Therefore, the identification of such modifiers and epigenetic adjusters among these accessions will help improve the efficiency of CRISPR/Cas-mediated genome editing possibly in CRISPR/Cas-resilient crops for trait improvement.

The isolated *cbf123* triple mutant in the C24RDLUC background (*cbf123LUC-2*) showed no transcripts of *CBF1*, *2*, and *3* genes and their protein products, indicating that this *cbf123LUC-2* mutant is a null mutant (**Figures 6A,B**). The previously reported *cbf* triple mutants showed different morphological phenotypes; Zhao et al found smaller sized *cbf* triple mutant (*cbf123-1*, *cbf123-2*) than Col-0, while Jia et al observed no difference in growth between Col-0 and their *cbf* triple mutant (*cbfs-1*) (Jia et al., 2016; Zhao et al., 2016). Our *cbf123LUC-2* showed a smaller size than its background line (C24RDLUC) at the 20-day-old stage. It should be noted that Jia et al. (2016) generated the *cbfs-1* mutant from *cbf3* T-DNA mutant. Therefore, it is tempting to speculate that *cbf3* T-DNA line might pose a different accession. Despite this uncertainty, the results from us and Zhao et al. suggest functions of *CBF1*, *CBF2*, and *CBF3* genes in growth and development at normal temperature.

In summary, we found that CRISPR/Cas system has different efficiency in different Arabidopsis accessions. Our results imply the existence of genetic modifiers and/or chromatin access difference for CRISPR/Cas-mediated genome editing in different plant accessions.

## Author Contributions

J-KZ, D-JY, and B-hL conceived and designed the research. SC, S-iY, JP, and YM performed the experiments. SC, S-iY, YM, J-KZ, D-JY, and B-hL discussed the results. SC, S-iY, and B-hL wrote the paper. SC and S-iY equally contributed to the work and should be regarded as joint First Authors.

## Conflict of Interest Statement

The authors declare that the research was conducted in the absence of any commercial or financial relationships that could be construed as a potential conflict of interest. The reviewer SX declared a shared affiliation and past co-authorship with one of the authors J-KZ to the handling Editor.

## Footnotes

1 http://www.genome.jp/tools-bin/clustalw

2 http://www.genome.jp/tools-bin/clustalw

3 http://crispr.hzau.edu.cn/CRISPR/

## Supplementary Material

**FIGURE S1 |** Schematic drawing of the CRISPR/Cas9 construct generation.

**FIGURE S2 |** Schematic drawing of the homozygous *CBF123* deletion in each accession.

**FIGURE S3 |** Anti-CBF123 antibody generation and activity. **(A)** CBF2 protein detection by anti-CBF123 antibody. A serial dilutions of CBF2-GST extracts from *E. coli* (left) and plant protein extract from Arabidopsis Columbia (right) were loaded and proteins were detected by anti-CBF123 antibody. Red arrows indicate the expected protein size. For anti-CBF123 antibody generation, cDNA fragment corresponding to the coding region of *CBF2* was amplified by PCR with primers containing attB1, attB2 sequence and cloned into donor vector (pDONR-zero) and subsequently moved into pDEST15 vector to produce recombinant protein with a GST protein The CBF2-GST recombinant vector was introduced into the BL21 strain of *Escherichia coli*. The *E. coli* transformants were grown at 37°C (OD600 = 0.8) and CBF2-GST was induced by 0.5 mM isoprophyl1-thio-β-D-galactopyranoside (IPTG). After 3 h at 30°C, cultures were harvested, resuspended in 1× PBS and incubated for 30min in the presence of 1% (v/v) Triton X-100 followed by disruption by sonication. The recombinant CBF2-GST fusion protein was purified by 12% SDS-PAGE and electro-eluted by use of Electro-Eluter (Model 422, BIO-RAD). The 500 mg of eluted CBF2 recombinant proteins were emulsified with the equal volume of Freund’s complete adjuvant and was injected subcutaneously at three sites in the rabbits’ backs. Two weeks later, the rabbits were injected with the 500 mg of CBF2 protein with Freund’s incomplete adjuvant as a booster immunization. A total of three booster injections were performed at 2-week intervals. One week following the final immunization, the antiserum was collected and stored at -20°C. **(B)** Cross-interaction of CBF123 proteins with anti-CBF123 antibody. Cross-interactions of the generated anti-CBF antibody were tested with Arabidopsis cbf2 or cbf3 single mutant (left) and transient expression of HA-tagged, CBF1-HA, CBF2-HA, and CBF3-HA proteins in tobacco leaves (right). The results suggest that the CBFs antibody could cross-interact with all CBF123 proteins. Red arrows indicate the expected protein size.
