# Supplementary figures and images for: Accession-Dependent CBF Gene Deletion by CRISPR/Cas System in Arabidopsis
Source: Front Plant Sci. 2017 Nov 7;8:1910. doi: 10.3389/fpls.2017.01910 (PMC5682037; doi:10.3389/fpls.2017.01910)

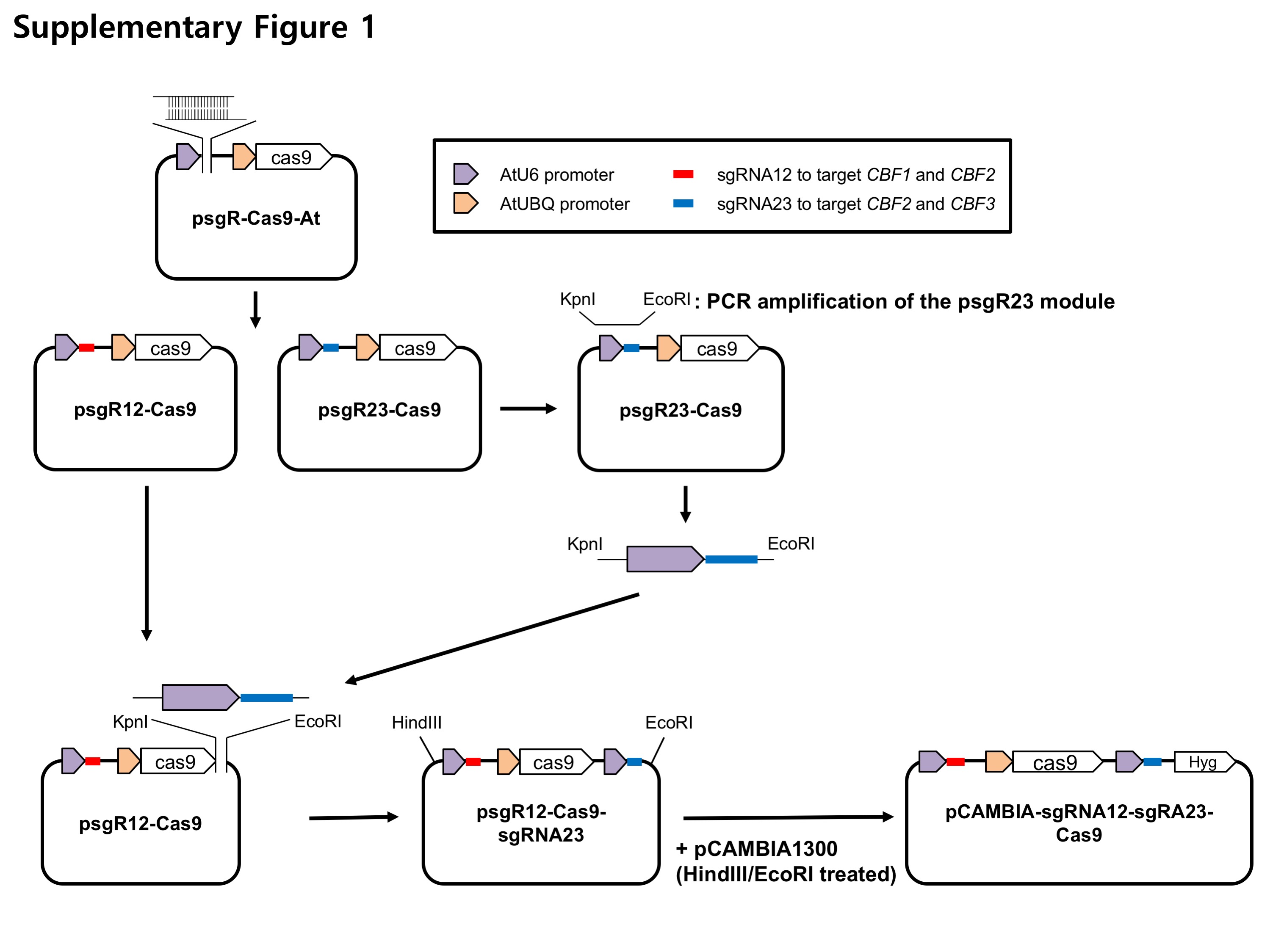

Supplement: Supplementary file 1 [file Image_1.JPEG]

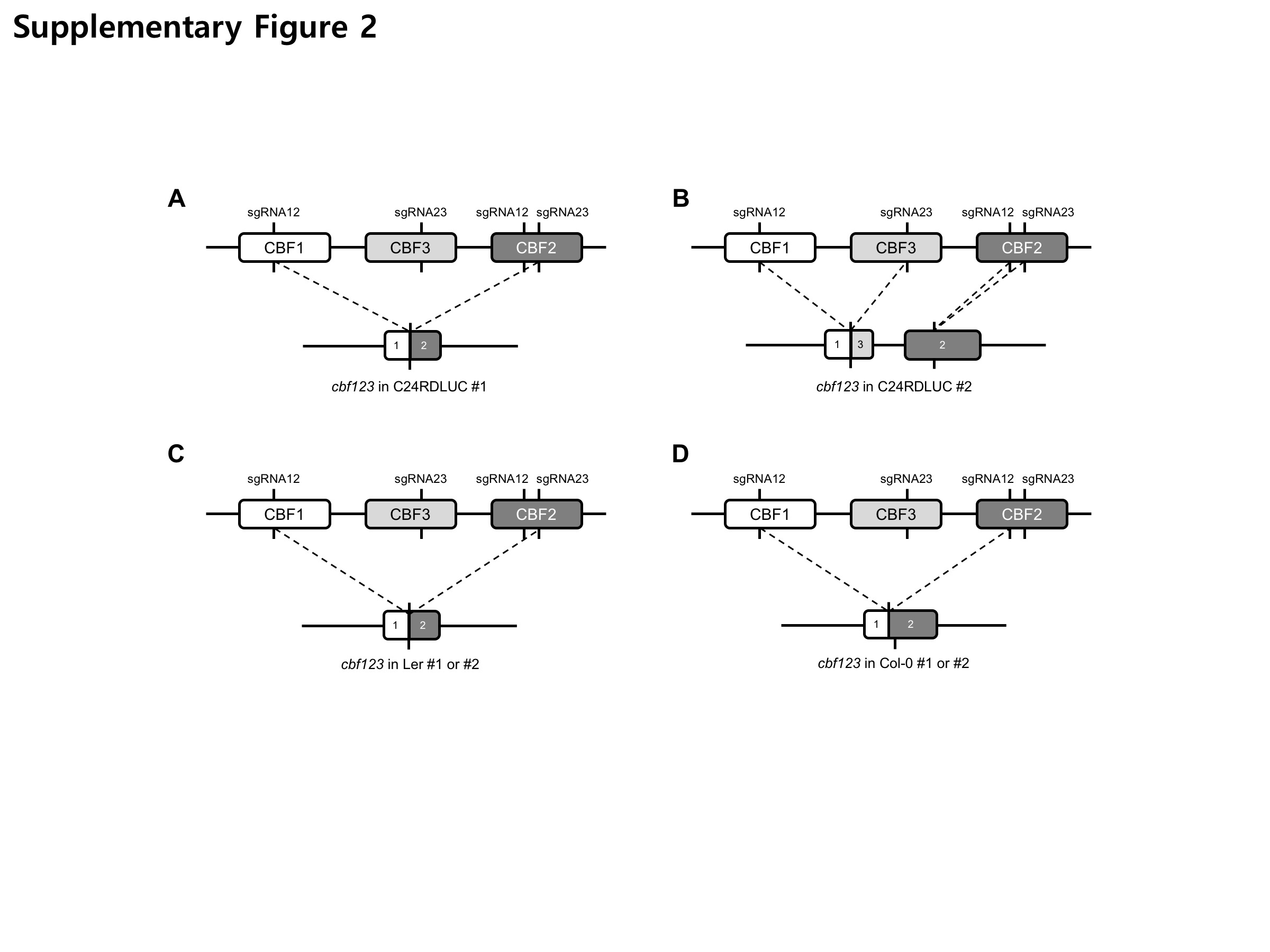

Supplement: Supplementary file 2 [file Image_2.jpeg]

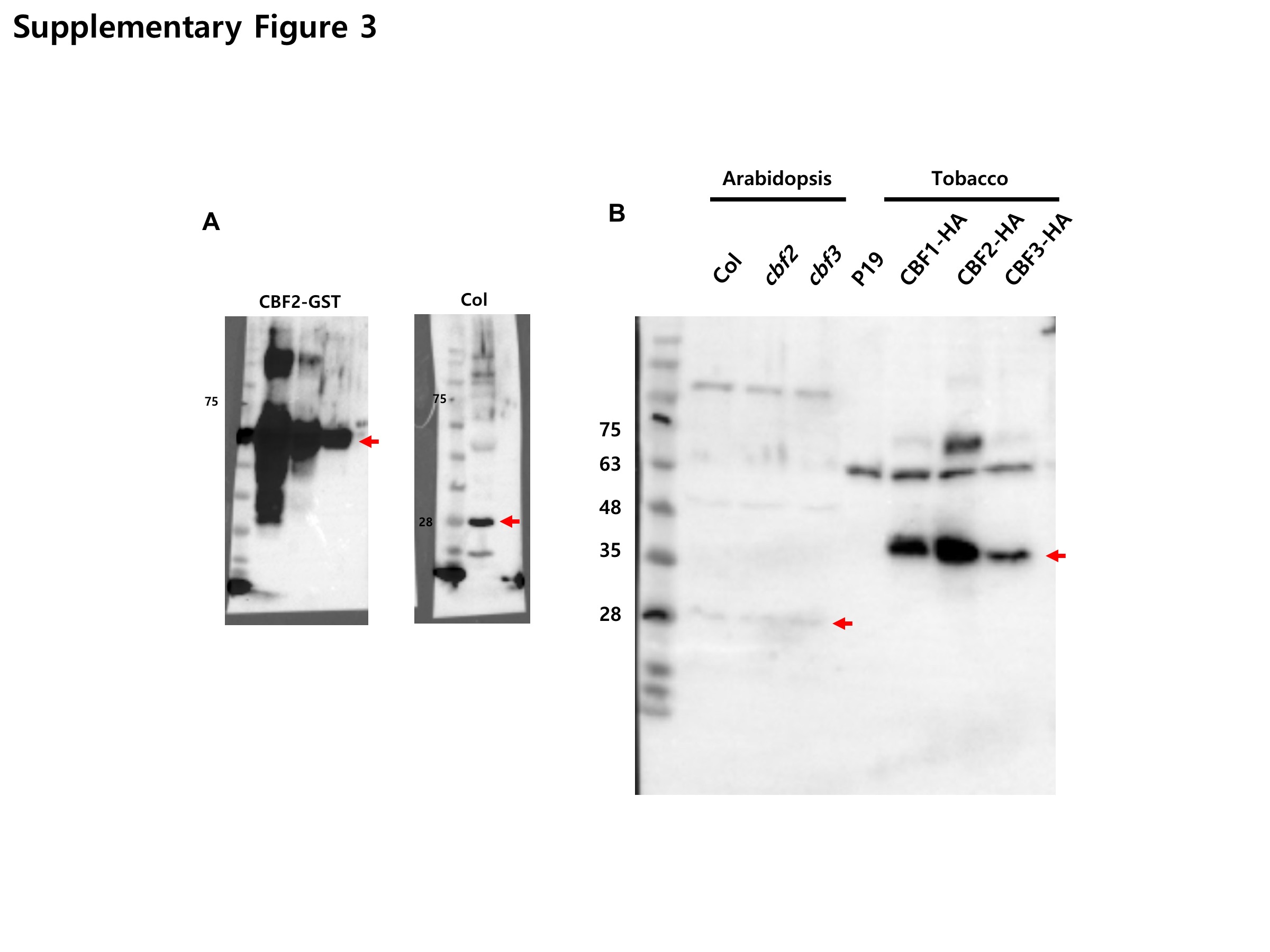

Supplement: Supplementary file 3 [file Image_3.jpg]
