# Supplementary material for: Accession-Dependent CBF Gene Deletion by CRISPR/Cas System in Arabidopsis
Source: Front Plant Sci. 2017 Nov 7;8:1910. doi: 10.3389/fpls.2017.01910 (PMC5682037; doi:10.3389/fpls.2017.01910)
Supplement: Supplementary file 4 [file Table_1.PDF]

Supplementary Table 1. *CBF123* deletion ratio in Col-0

Col-0 T1 #	*CBF123* deletion in T1*	T2		
		Number of plants with *CBF123* deletion	Number of plants examined	% of *CBF123* deleted plants
1	Y	10	24	41.67
3	N	1	24	4.17
4	Y	15	24	62.50
5	N	20	24	83.33
6	N	10	24	41.67
7	Y	20	24	83.33
8	Y	10	24	41.67
9	N	14	24	58.33
10	Y	3	24	12.50
11	Y	5	24	20.83
13	Y	7	24	29.17
14	N	15	24	62.50
15	Y	11	24	45.83
16	N	0	9	0.00
17	Y	4	24	16.67
19	Y	0	24	0.00

*, Y = *CBF123* deleted, N = *CBF123* not deleted
