# Supplementary material for: Accession-Dependent CBF Gene Deletion by CRISPR/Cas System in Arabidopsis
Source: Front Plant Sci. 2017 Nov 7;8:1910. doi: 10.3389/fpls.2017.01910 (PMC5682037; doi:10.3389/fpls.2017.01910)
Supplement: Supplementary file 5 [file Table_2.PDF]

Supplementary Table 2. *CBF123* deletion ratio in C24RDLUC

C24RDLUC T1 #	*CBF123* deletion in T1*	T2		
		Number of plants with *CBF123* deletion	Number of plants examined	% of *CBF123* deleted plants
3	Y	10	24	41.67
5	N	0	21	0.00
11	N	1	24	4.17
12	N	0	12	0.00
13	N	0	2	0.00
14	Y	8	15	53.33
15	Y	11	24	45.83
16	Y	0	24	0.00
19	N	0	11	0.00
20	Y	9	24	37.50
21	Y	10	24	41.67
24	Y	3	24	12.50
25	Y	0	2	0.00
29	N	8	17	47.06
30	N	1	24	4.17
31	Y	0	13	0.00
33	N	0	24	0.00
34	Y	16	24	66.67
37	Y	0	20	0.00
38	N	1	17	5.88
41	Y	0	9	0.00
42	Y	16	46	34.78
47	N	2	22	9.09
48	N	0	24	0.00
49	Y	1	5	20.00
50	Y	78	89	92.13
51	Y	2	11	18.18

*, Y = *CBF123* deleted, N = *CBF123* not deleted
