# Supplementary material for: Accession-Dependent CBF Gene Deletion by CRISPR/Cas System in Arabidopsis
Source: Front Plant Sci. 2017 Nov 7;8:1910. doi: 10.3389/fpls.2017.01910 (PMC5682037; doi:10.3389/fpls.2017.01910)
Supplement: Supplementary file 6 [file Table_3.PDF]

Supplementary Table 3. *CBF123* deletion ratio in Ler

Ler T1 #	*CBF123* deletion in T1*	T2		
		Number of plants with *CBF123* deletion	Number of plants examined	% of *CBF123* deleted plants
1	Y	0	24	0.00
2	Y	0	24	0.00
3	Y	0	24	0.00
4	Y	0	24	0.00
5	N	0	24	0.00
7	N	0	4	0.00
8	Y	0	20	0.00
11	Y	0	24	0.00
16	Y	0	24	0.00
17	N	1	24	4.17
18	N	0	24	0.00
19	N	0	24	0.00
20	Y	0	24	0.00
21	N	0	24	0.00
22	N	0	24	0.00
23	Y	0	8	0.00
24	N	0	7	0.00
35	Y	0	24	0.00
36	N	0	24	0.00
37	Y	6	24	25.00
39	N	7	24	29.17
40	N	0	24	0.00
41	Y	6	24	25.00
42	N	0	24	0.00
43	N	0	19	0.00
44	Y	6	24	25.00
45	N	0	24	0.00
47	N	0	24	0.00
48	N	0	24	0.00

*, Y = *CBF123* deleted, N = *CBF123* not deleted
